# Evaluation of Cellulolytic Endo-1,4-β-D-Glucanase Activity in the Digestive Fluid of Adult Phytophagous Beetle *Hoplasoma unicolor*

**DOI:** 10.21315/tlsr2021.32.3.4

**Published:** 2021-09-30

**Authors:** Mohammad Mosleh Uddin, Suzana Afrin Lima, Tanim Jabid Hossain, Newton Kar, Yeasmin Zahan, Babamale AbdulKareem Olarewaju

**Affiliations:** 1 Department of Biochemistry and Molecular Biology, Mawlana Bhashani Science and Technology University, Santosh, Tangail 1902, Bangladesh; 2 Department of Biochemistry and Molecular Biology, University of Chittagong, Chittagong 4331, Bangladesh; 3 Parasitology Unit, Department of Zoology, University of Ilorin, Ilorin 1515, Nigeria

**Keywords:** Cellulolytic Activity, Endo-1, 4-β-D-Glucanase, Beetle, Insect Gut Fluid, Insect Cellulase, Carboxymethyl Cellulose, Zymography

## Abstract

Insects of the taxonomic order Coleoptera are recognised for considerable cellulolytic activity in their digestive fluid. The cellulolytic activity of the gut fluid in *Hoplasoma unicolor*, a member of Coleoptera, however, remains unexplored. In this study, we, for the first time, report the qualitative and quantitative analysis of cellulolytic activity in the digestive fluid of this insect. The cellulolytic endo-1,4-β-D-glucanase activity was confirmed in the supernatant of the insect’s digestive fluid by agar plate assay using carboxymethyl cellulose as the substrate. To determine the optimum pH, enzyme activity was further assessed in an acidic pH range of 5 to 6, and the highest activity was observed at pH 5.3. For quantitative analysis, endoglucanase activity was measured using 3,5-dinitrosalicylic acid method which revealed that the specific activity of the gut sample was 0.69 (±0.01) units per mg of protein. For further characterisation of the cellulases in the sample, SDS-PAGE and zymogram analysis were carried out. Two active cellulolytic bands were detected on the zymogram suggesting the presence of two distinct endoglucanases which completely disappeared upon heating the sample at 55°C. Our study, therefore, highlights prospect of the gut fluid of *H. unicolor* as an important source of cellulase enzymes that merits further investigations into their extensive characterisation for potential industrial applications.

**Highlights**

- Presence of cellulolytic endoglucanase activity was detected in digestive fluid of the beetle *Hoplasoma unicolor*.
- Quantitative estimation showed a relatively high cellulase activity with specific activity measured as 0.69 (±0.01) units per mg of protein.
- Zymogram analysis revealed the presence of two active cellulolytic proteins in the gut extract.

## INTRODUCTION

Cellulases are hydrolytic enzymes that catalyse the cleavage of 1,4-β-glycosidic bonds between glucose residues present in cellulose, the most abundant biopolymer produced on earth and the major constituent of agricultural and industrial wastes (Bayer *et al*. 1998; Dashtban *et al*. 2010). Consequently, substantial efforts are being made during the past few decades into prospecting for novel cellulolytic enzymes and elucidation of their catalytic properties so that the enzymes could be employed in many important bioprocesses, most particularly in: (1) the production of cost-effective and sustainable biofuels from the cellulosic and lignocellulosic biomass, (2) efficient and ecofriendly management of waste disposal and (3) industrial processes specially the textile, paper, food and detergent industries (Bayer *et al*. 2007; Jayasekara & Ratnayake 2019; Phitsuwan *et al*. 2013). The cellulase enzymes refer to three distinct types of cellulolytic hydrolases, e.g., endo-1,4-β-D-glucanase (endoglucanase; EC 3.2.1.4), exo-1,4-β-D-glucanase (cellobiohydrolase; EC 3.2.1.91 and 3.2.1.176), and β-glucosidase (EC 3.2.1.21) (Okano *et al*. 2014). The endoglucanase catalyses hydrolysis of soluble and insoluble β-(1,4)-glucan substrates, either directly on the polymer and/or shorter (poly)-oligosaccharides, both internally as well as from reducing and nonreducing ends in a nonprocessive or processive manner to produce oligosaccharides of various lengths (Girfoglio *et al*. 2012; Hobdey *et al*. 2015). Exoglucanases progressively hydrolyse cellulose at the reducing and non-reducing ends to release cellobiose moieties. Whereas β-glucosidases catalyse the final step: hydrolysis of the products generated by both endo- and exo-glucanases, i.e., soluble cellulodextrins and cellobioses to produce glucose. Although complete degradation of cellulose into glucose requires synergistic action among the three hydrolases, endoglucanase is believed to be the most important of the three (Annamalai *et al*. 2016). These cellulolytic hydrolases are commonly referred to as carboxymethylcellulase or CMCase, since carboxymethylcellulose (CMC) is the substrate most widely used for the determination of functional cellulase activity in experimental procedures (Ali *et al*. 1995).

The endoglucanase enzymes characterised so far are mostly those of microbial and plant origin. This enzyme activity has long been believed to be limited to only bacteria, fungi and plants. While activity was also detected in the digestive fluid of lower animals such as the insects, it was totally attributed to the microbial symbionts in the insect gut until 1998 when Watanabe *et al*. first described the identification of an endo-1,4-β-D-glucanase gene in the termite *Reticulitermes speratu*. Today, insects from at least 20 families such as Acrididae, Buprestidae, Amphisbatidae, Tenthredinidae, etc. are known to produce their own cellulolytic enzymes (Su *et al*. 2013). Insects, therefore, are regarded as very promising candidates to search for novel cellulases. The highly adapted phytophagous insects appear to be the most important species in this regard since they feed on very fibrous and lignocellulose-rich plant tissues (Oppert *et al*. 2010).

In the present study we sought to evaluate cellulolytic activity in gut fluid of the beetle *Hoplasoma unicolor*, a phytophagous insect of the taxonomic order Coleoptera. Previously, a number of beetles have been shown to possess considerable amount of cellulolytic activity in their gut or head fluid (Table 1). Su *et al*. (2013), for example, investigated cellulolytic activities in the gut fluids of 54 insect species from seven orders and detected highest CMCase activities in the insects of Coleoptera and Orthoptera. Other groups also reported high endoglucanase activity in the gut fluids of several other beetle species (Table 1). Beetles, therefore, are considered to be attractive candidates to prospect for novel cellulolytic enzymes with remarkable catalytic potential.

We herein report the determination of endo-1,4-β-D-glucanase activity in the digestive fluid of adult *H. unicolor*, a beetle that feeds on foliage of herbs (Mathew *et al*. 2005). This beetle was found abundantly feeding on its host plant, *Clerodendrum villosum*, a tomentose shrub (Hazmi *et al*. 2019). To our knowledge, cellulolytic activity in the gut fluid of this beetle was not reported previously. Therefore, qualitative and quantitative measurement of the enzyme activity was carried out on CMC using the partially purified gut fluid prepared from this insect. SDS-PAGE and zymogram analyses were also performed to further understand the multiplicity of the active cellulolytic proteins present in the enzyme extract.

## MATERIALS AND METHODS

### Insect Collection

**Figure 1:**
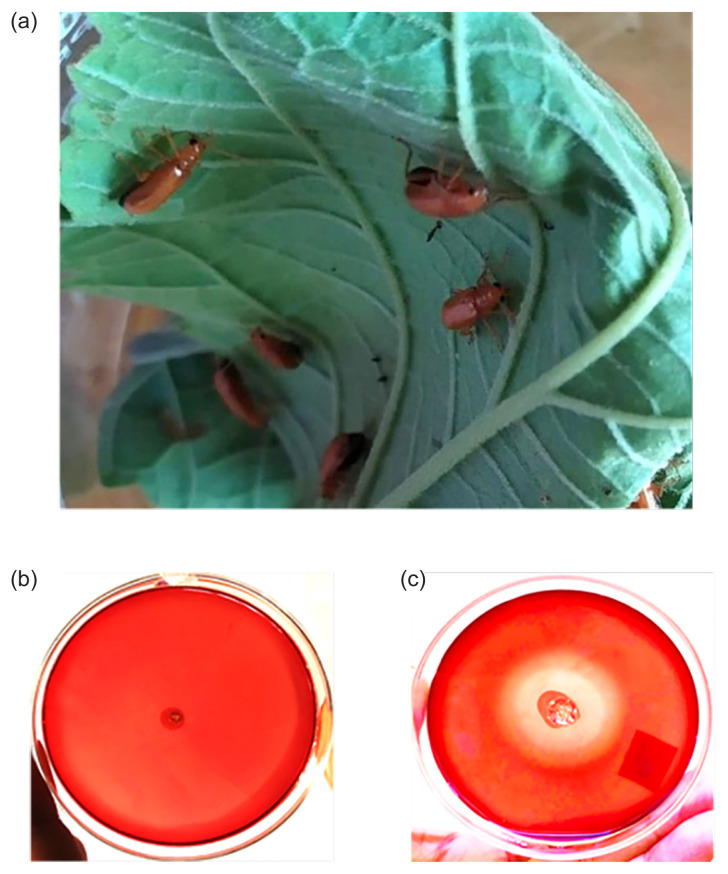
Endoglucanase activity of the digestive fluid from *H. unicolor* adults. (a) Beetles on its host plant at the time of collection; (b) and (c) Screening endo-1,4-β-D-glucanase activity in the gut extract prepared from digestive fluid of the beetle. Wells were made in agar media containing CMC and loaded with either buffer solution (b) or the gut extract (c). Clear zone was only produced by the gut extract indicating the presence of endoglucanase activity.

This study investigated cellulolytic activity in the gut fluid of the beetle *H. unicolor*. Several beetles are already known to possess notable amount of cellulase enzymes in their gut fluid. Being a phytophagous beetle, *H. unicolor* is also speculated to produce considerable amount of cellulase enzymes in its gut which still remains unstudied. Hence in the present study, twenty adult beetles, ~1.2 cm in length, were collected from the host plant *C. villosum* near the campus of Mawlana Bhashani Science and Technology University, Bangladesh in the month of September 2019. The insects were actively feeding on or in close proximity to the host plant at the time of collection (Fig. 1a). Insects were kept on the leaves during transportation to the laboratory and placed in ice for 10 min before dissection.

### Dissection and Preparation of Digestive Extract for Enzyme Assays

Dissections of the insects were performed on ice. Insect guts were removed and collected in microcentrifuge tube emerged in ice. The dissected guts were combined, cut into small pieces, homogenised by vortexing and centrifuged at 10,000 rpm for 10 min at 4°C. The 130 μL of supernatant was collected and transferred to a fresh microcentrifuge tube and stored at −20°C until it is ready to use. This gut sample has been designated hereafter as gut extract or enzyme extract.

### Qualitative Endoglucanase Assay

Cellulolytic activity of the gut extract was determined essentially as described previously (Hossain *et al*. 2020). Briefly, 100 μL of the gut extract was placed inside wells in agar media (pH 5, 5.3 and 6) supplemented with 1% (w/v) CMC (Sigma-Aldrich) in 0.1 M sodium acetate buffer. A control plate was also included where gut extract was replaced with the same volume of buffer only. After overnight incubation at 37°C, the plates were stained with 0.1% Congo red solution for 5–10 min and destained with 0.1M NaCl for 10–15 min. Formation of clear zone was considered positive result for endoglucanase activity.

### Protein Estimation

Amount of proteins in the gut extract was estimated by Lowry protein assay method using bovine serum albumin (BSA) as standard (Lowry *et al*. 1951).

### Electrophoresis

The enzyme extract was examined by sodium dodecyl sulphate-polyacrylamide gel electrophoresis (SDS-PAGE) following the method as described by Laemmli (1970). Briefly, aliquots containing samples equivalent to 0.8 μg and 4 μg of protein were applied to pre-casted gel and electrophoresis was conducted with running buffer (25 mM Tris, 192 mM glycine with 0.1% SDS) at constant voltage of 110 V. Proteins were stained with Coomassie Brilliant Blue R-250.

### Zymogram Analysis

We carried out zymogram analysis to detect the specific proteins having cellulolytic activity in the gut extract as described in a previous work (Schwarz *et al*. 1987) with small modifications as in Uddin *et al*. (2012). Gel was prepared including 0.1% CMC before polymerisation of resolving gel. Gut sample (0.8 μg or 4 μg of protein) containing 1× loading buffer (50 mM Tris HCl pH 6.8, 1.5% SDS, 0.02% bromophenol blue, 10% glycerol and 2% β-mercaptoethanol) was loaded onto the gel, with or without a pre-heat treatment at 55°C for 30 min. Electrophoresis was conducted at 4°C at 110 V. After electrophoresis, gel was washed five times (5–6 min each) in 50 mL of washing buffer (0.1 M sodium succinate pH 5.3) at room temperature followed by a final wash for 30 min with the same buffer. Gel was stained with 0.1% Congo red solution for 10–15 min and destained using 1 M NaCl until clear depolymerisation bands became visible.

### Quantitative Endoglucanase Assay

Cellulase activity was quantified using a modified 3,5-dinitrosalicylic acid (DNSA) assay (Miller 1959). Enzyme extract (20 μg) was mixed with 235 μL CMC (1% CMC sodium salt in 0.1 M Na-acetate buffer pH 5.3). After incubation at 37°C for 45 min in water bath, 450 μL of DNS was added to stop the reaction followed by heating in boiling water bath for 10 min and then 40% of 230 μL of Rochelle salt was added. Absorbance was measured at 540 nm by UV spectrophotometer against a reaction blank in which DNS solution was added before the enzyme extract. A standard curve of absorbance against glucose (50–400 μg) was constructed to calculate the amount of reducing sugar released (glucose equivalents) during the assay. One unit of cellulolytic activity was defined as the amount of enzyme required to produce 1 μmol of reducing sugar (glucose equivalents) per min at 37°C at pH 5.3. Specific activity is described as units per mg of protein. The experiment was done in triplicate and the mean value is presented.

## RESULTS AND DISCUSSION

### Endo-1,4-β-D-Glucanase Activity of the Gut Extract

This work aimed at assessing the endo-1,4-β-D-glucanase activity in digestive fluid of the phytophagous beetle *H. unicolor*. To this end, we collected digestive fluid from 20 adult insects and obtained its protein fraction by centrifugation. This partially purified gut extract was examined for the presence of endoglucanase activity in an agar plate assay using CMC as the substrate. A distinct zone of clear halo was formed in the agar media when spotted with the gut extract that clearly indicated depolymerisation of CMC in the media confirming presence of cellulolytic proteins in the sample [Figs. 1(a) and 1(b)]. With several beetles from the order Coleoptera are already known to have cellulolytic activity in its digestive fluid (Table 1), our study, therefore, makes a new inclusion, *H. unicolor*, to that list. Although most of the previous studies didn’t confirm whether the respective enzymes are encoded within the insect genome or of microbial origin (Geib *et al*. 2010; Oppert *et al*. 2010; Rehman *et al*. 2009; Sami & Shakoori 2008; Su *et al*. 2013; Uddin *et al*. 2012). In our study as well, whether the endoglucanase activity is endogenous or, secreted from microbial symbionts, or, contributed by both, needs to be investigated by more extensive analysis in the future. In the digestion of recalcitrant plant matters, enzymes from both the insects and the symbionts are required for complete breakdown of the polymers, e.g., cellulose into simple sugars (Shelomi *et al*. 2020). Availability of the genome sequence of the beetle might also provide us gaining more insights into its endogenous cellulases.

In previous works, the endoglucanase activity assay of the gut fluid of Coleoptera insects was mostly carried out at pH ~5 to 6 (Geib *et al*. 2010; Oppert *et al*. 2010; Su *et al*. 2013; Vatanparast *et al*. 2014; Willis *et al*. 2010). Whereas in a study with *Podontia quatuordecimpunctata*, an insect from the same family as *H. unicolor*, the enzyme assay was also performed at pH 5.3 (Uddin *et al*. 2012). Therefore, to determine the optimum pH for endoglucanase activity of the enzyme extract, we initially decided to perform the agar plate assay at three different pH ranging from 5 to 6. Area of clear halos formed on the agar plates, which indicates the enzyme intensity (Hossain *et al*. 2020), was visually inspected. We found that the endoglucanase activity of the gut fluid was highest at pH 5.3, whereas the activity decreased below and above this pH (i.e., pH 5 and 6) with the smallest zone of clearance formed at pH 6. Since lower enzyme activity was found on either side of pH 5.3, therefore, the endoglucanase activity was not examined at other pH ranges. Hence, it appears from the above findings that the cellulolytic enzymes present in the gut sample exert maximum activity at acidic pH. This finding is consistent with those reported in a number of previous research. For example, the endoglucanses from gut fluid of a number of beetles and termites as well as bacteria and fungi showed highest activity at slightly acidic pH mostly in the range of pH 5 to 6 (Busch *et al*. 2018; Hatefi *et al*. 2017; Tokuda *et al*. 1997; Zhang *et al*. 2011). Insect cellulases with optimum activity at alkaline pH were also reported though (Sami & Shakoori 2008; Willis *et al*. 2011).

### SDS-PAGE and Zymogram Analysis

**Figure 2:**
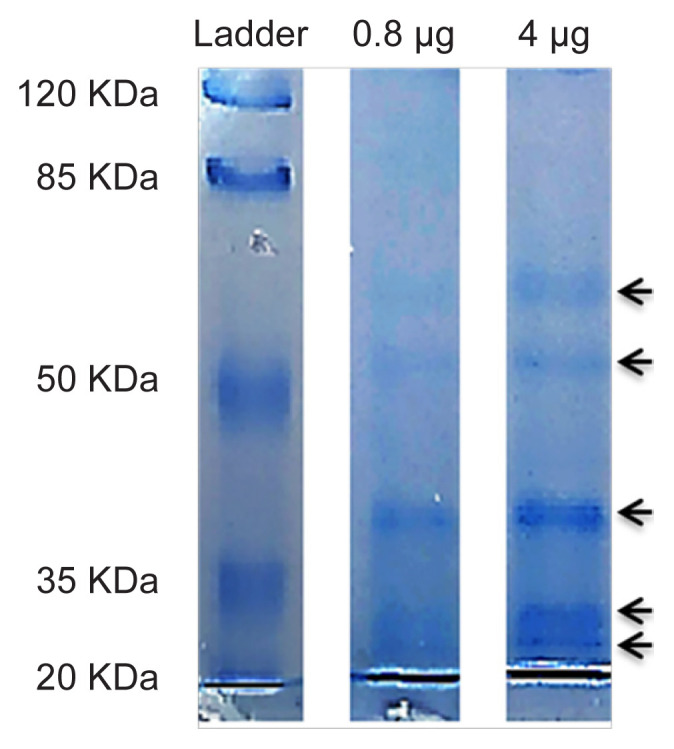
Separation of proteins of the gut extract by SDS-PAGE. Samples containing 0.8 μg or 4 μg of proteins were loaded onto the gel and, after electrophoresis, stained with Coomassie Brilliant Blue. Each of the protein bands detected is indicated by an arrow-head.

SDS-PAGE analysis of the digestive extract was carried out to examine the purity and number of proteins present in the partially purified extract and to reveal their molecular weight (MW). The gut extract was used at two different concentrations, 0.8 μg or 4 μg of protein, in the analysis. Five distinct protein bands were detected at both concentrations with the MW ranging from ~25 to 65 kDa (Fig. 2), suggesting that the MW of the cellulolytic proteins in the sample lies within this range. Detection of only five protein bands also indicates that the gut sample could be fairly purified.

**Figure 3:**
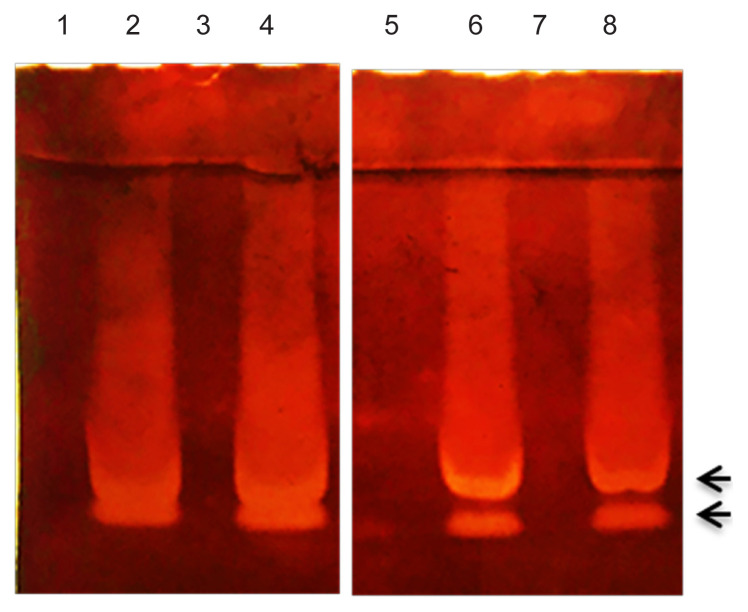
Zymogram analysis for detection of the active endoglucanases in the digestive fluid of adult *H. unicolor*. Proteins in the sample (~800 ng) were separated by electrophoresis on gel containing CMC, and the active endoglucanases were subsequently visualised by staining with Congo red. Clear bands in the gel indicate cellulolytic activity due to the degradation of CMC. Arrow-heads indicate the position of the active enzymes. Lanes 1 and 3: 4 μg sample pre-heated at 55°C. Lanes 2 and 4: 4 μg sample without pre-heat treatment. Lanes 5 and 7: 0.8 μg sample pre-heated at 55°C. Lanes 6 and 8: 0.8 μg sample without pre-heat treatment.

To find out which of the five proteins were the active endoglucanase enzymes in the digestive extract, we performed a zymogram analysis using CMC as the substrate. Two prominent bands of clearance due to the degradation of CMC were observed that were in close proximity to each other which suggested the presence of at least two endoglucanase enzymes in the gut sample (Fig. 3). When the sample was pre-heated at 55°C for 30 min before loading onto the gel, no hydrolysis band was detected indicating complete loss of enzyme activity due to heat inactivation; although a number of insect cellulases were previously demonstrated to be enzymatically active at similar temperatures (Sami & Shakoori 2008; Tokuda *et al*. 1997; Watanabe *et al*. 1997). Together, the results of SDS-PAGE and zymogram analysis suggested that only two of the five proteins detected in the gut sample possessed endoglucanase activity in the experimental conditions used. Detection of multiple cellulase enzymes in insect gut sample is not uncommon. Similar to our findings, two cellulolytic protein bands were reported in other insects such as *Tribolium castaneum*, *Syrbula admirabilis* and *Tenebrio molitor* (Oppert *et al*. 2010; Rehman *et al*. 2009). Some studies also described the detection of more than two cellulolytic proteins in zymography (Su *et al*. 2013; Uddin *et al*. 2012; Willis *et al*. 2010 ).

The two active enzymes detected in the zymography were very close to each other having similar molecular masses. Of the five proteins detected in SDS-PAGE, on the other hand, only the two at bottom of the gel were closely placed. Taken together, we assume that the proteins which were found in close vicinity in SDS-PAGE analysis with MW of ~25 and ~30 kDa, might be the endoglucanases. The MW of these two enzymes is similar to those reported in various other insects as well as in other organisms. For example, *Rhagium inquisitor*, *Dissosteira carolina*, *S. admirabilis*, *T. molitor* and others were documented to have cellulases in their digestive fluids with the MW lying in the range of 20 to 35 kDa (Oppert *et al*. 2010; Rehman *et al*. 2009; Willis *et al*. 2010).

### Quantitation of Endo-1,4-β-D-Glucanase Activity

Finally, to determine specific activity, we carried out a quantitative analysis of endoglucanases present in the enzyme extract towards CMC. The specific enzyme activity was measured as 0.69 (± 0.01) U/mg of protein which seems quite high as compared to those reported in the insects of the same order (Table 1) and in other insects as well (Oppert *et al*. 2010). The endoglucanase activity in the members of Coleoptera has been reported to vary over a very wide range, from as little as 0.01 to as much as 2.80 U/mg (Table 1). The highest activity was found in the beetle *Morimus funereus* (Dojnov *et al*. 2013). Direct comparison of the activities among various endoglucanases is not, however, fully coherent due to the different experimental conditions used in the studies.

## CONCLUSION

Findings of this paper represent the first description and preliminary characterisation of the cellulolytic activity in the digestive fluid of *H. unicolor*. The endo-1,4-β-D-glucanases of the gut fluid showed prominent efficiency in digestion of cellulose with specific activity higher than those of most other insects. Further research for purification and complete characterisation of the endoglucanases are, therefore, needed to understand their origin and catalytic efficacy which can provide foundation for development of an efficient and inexpensive system for biofuel production and waste management based on the hydrolytic enzymes of insect digestive fluid.

## Figures and Tables

**Table 1:** Endo-1,4-β-D-glucanase activity analysed in beetles of the taxonomic order Coleoptera.

Family	Name of species	Life stages	Body segment	Substrate^s^	Specific activity^a^ (U/mg)	References
Cerambycidae	*Morimus funereus*	Larva	G	CMC	1.20	Dojnov *et al*. 2013)
		Larva^d1^	G	CMC	2.40	
		Larva^d2^	G	CMC	2.80	
		Larva^d3^	G	CMC	2.80	
	*Anoplophora glabripennis*	Adult^d4,m^	G	CMC	15.49^u^	(Li *et al*. 2008)
		Adult^d4,f^	G	CMC	9.61^u^	
		Adult^d5,m^	G	CMC	13.06^u^	
		Adult^d5,f^	G	CMC	10.34^u^	
		Adult^d6,m^	G	CMC	13.54^u^	
		Adult^d6,f^	G	CMC	8.02^u^	
		Adult^d7,m^	G	CMC	17.12^u^	
		Adult^d7,f^	G	CMC	10.76^u^	
	*Cerambycid* sp.	Adult	G	CMC	0.29	(Shi *et al*. 2011)
		Larva	G	CMC	0.17	(Oppert *et al*. 2010)
				MCC	0.03	
	*Elaphidion mucronatum* (Say)	Larva	G	CMC	0.42	
				MCC	0.025	
	*Neoclytus a. acuminatus* (Fabricius)	Larva	G	CMC	0.35	
				MCC	0.08	
	*Elaphidion mucronatum*	Larva	G	CMC	0.42	
				MCC	0.025	
	*Megopis sinica*	Larva	G	CMC	0.53	(Su *et al*. 2013)
				FP	0.21	
	*Trichoferus campestris*	Larva	G	CMC	0.22	
				FP	0.03	
	*Batocera horsfieldi*	Larva	G	CMC	0.21	
				FP	0.008	
	*Massicus raddei*	Larva	G	CMC	0.27	
				FP	0.01	
	*Anoplophora glabripennis*	Larva	G	CMC	0.41	(Geib *et al*. 2010)

Tenebrionidae	*Tribolium castaneum*	Adult	G	CMC	0.02	(Oppert *et al*. 2010)
			H	CMC	0.02	
				MCC	0.01	
					
	*Tenebrio molitor*	Larva	G	CMC	0.02	
			H	CMC	0.03	
			G	MCC	0.05	
			H	MCC	0.01	
	*Tribolium castaneum*	Adult	Whole insect	CMC	–	(Rehman *et al*. 2009)
	*Tribolium castaneum*	Larva	–	CMC	12.9^c^	(Willis *et al*. 2011)
	*Tenebrio obscurus*	Larva	G	CMC	0.07	(Su *et al*. 2013)
				FP	0.08	
	*Zophobas morio*	Larva	G	CMC	0.222	(Szentner *et al*. 2019)
		Adult	G	CMC	0.517	

Chrysomelidae	*Leptinotarsa decemlineata*	–	G	CMC	0.28	(Oppert *et al*. 2010)
			H	CMC	0.18	
			G	MCC	0.005	
			H	MCC	0.02	
	*Podontia quatuordecimpunctata*	Larva	G	CMC	1.73	(Uddin *et al*. 2012)
	*Aulacophora foveicollis*	Larva	G	CMC	–	(Sami & Shakoori 2008)
	*Xanthogaleruca luteola*	Larva	G	CMC	–	(Vatanparast *et al*. 2012)
	*Gastrophysa viridula*	Larva	G	CMC	–	(Busch *et al*. 2018)
		Adult	G	CMC	–	
	*Hoplasoma unicolor*	Adult	G	CMC	0.69	Present study

Curculionidae	*Rhynchophorus ferrugineus*	Larva	G	CMC	–	(Vatanparast *et al*. 2014)
	*Graphognathus leucoloma*	Larva	G	CMC	0.27	(Oppert *et al*. 2010)
				MCC	0.02	

Scarabaeidae	*Phyllophaga* sp	Larva	G	CMC	0.01	(Oppert *et al*. 2010)
			H	CMC	0.01	
			G	MCC	0.08	
	*Popillia japonica*	–	G	CMC	0.03	
				MCC	0.02	

Lyctidae	*Lyctus [prob. planicollis Lec.]*	Adult	G	CMC	0.20	(Oppert *et al*. 2010)
				MCC	0.06	

Scolytinae	*Scolytus* [prob. *rugulosus (Müller)*]	Larva	G	CMC	0.55	(Oppert *et al*. 2010)
				MCC	0.03	

Buprestidae	*Chrysobothris* sp.	Larva	G	CMC	0.29	

Lucanidae	*Dorcus titanus*	Larva	G	CMC	0.05	(Su *et al*. 2013)
				FP	0.03	

Anobiidae	*Lasioderma serricorne*	Larva	G	CMC	0.03	(Su *et al*. 2013)
				FP	0.015	
	*Leptinotarsa decemlineata*	Larva	G	CMC		
	*Lasioderma serricorne*	Larva	G	CMC		(Minoo *et al*. 2012)

Elateridae	*Pyrearinus termitilluminans*	Larva	G	CMC	0.014	(Colepicolo-Neto *et al*. 1986)

*Note*: d1 to d7 = Fed with experimental diets; m = Male, f = Female; s = CMC: CarboxyMethyl Cellulose, MCC: Micro Crystalline Cellulose, FP: Filter Paper; a = In most papers the specific activity was not exactly mentioned and the info was rather extracted from the figures; u = μmol (glucose)·g^−1^ (fresh weight)·h^−1^; c = Cellulase gene was cloned and expressed.
